# Toboggan or Not Toboggan

**Published:** 1987-11

**Authors:** M. J. Cobby, E. A. Thomas, A. H. Troughton

**Affiliations:** Radiology Department, Bristol Royal Infirmary, Bristol; Radiology Department, Bristol Royal Infirmary, Bristol; Radiology Department, Bristol Royal Infirmary, Bristol


### Toboggan or not Toboggan

**M. J. Cobby, E. A. Thomas, A. H. Troughton**
Radiology Department, Bristol Royal Infirmary, Bristol

### INTRODUCTION

It is well known that every year each flurry of snow brings elderly ladies in their droves to the Accident Department with their broken wrists and hips. At the same time young professionals fly abroad, and like lemmings, ski down the slopes straight to their hospital beds. What is not so well known is that their younger brothers and sisters take to the Bristol pistes themselves armed with fertiliser bags, tin trays and wooden sledges, ready to engage in an even more hazardous pursuit. From Brandon Hill to Ashton Court, Blaise Castle to Dundry Hill, the Sunday afternoon tranquillity is shattered by squeals of delight followed by groans of anguish from their kamikaze antics.

### Sunday 19 January 1986

For the resident radiology registrar at the Bristol Royal Infirmary it seemed to be another ordinary day; the Sunday newspapers had been read, a bout of fisticuffs with the surgeons narrowly averted, and all that was left to look forward to was 'Songs of Praise' in the evening. However, when the twelfth serious tobogganing injury had been reported, the registrar, alert as ever, began to spot a trend. "How many sledging incidents happen on a normal weekend?" he mused.

### The Damage

In all 23 people had injuries serious enough to warrant an X-ray. They aged between 4 and 39 years old, of whom nineteen were 16 years old or less. There were fifteen boys and eight girls. Table 1 shows the type of injuries sustained.

**Table 1**

| | *Hit by sledge* | *Fell off sledge* | *Hit obstacle* | *Total* |
|---|---|---|---|---|
| Fracture | 5 | 3 | 3 | 11 |
| Soft tissue injury | 4 | 4 | 4 | 12 |
| Total | 9 | 7 | 7 | 23 |

Although the number of fractures is distributed fairly evenly between the groups, the types of fractures differed significantly, as shown in table 2.

**Table 2**

| *Fracture* | *Hit by* | *Fell off* | *Hit obstacle* |
|---|---|---|---|
| Hand | 1 | 2 | 0 |
| Tibia | 3 | 0 | 0 |
| Ankle | 0 | 0 | 2 |
| Clavicle | 1 | 0 | 0 |
| Patella | 0 | 0 | 1 |
| Nose | 0 | 1 | 0 |

Table 2 shows that all the more serious injuries were caused, as might be anticipated, either by being hit by a sledge or by running into an inanimate object. Thus all three tibial fractures resulted from sledges running into people, and the two ankle and one patella fractures from hitting some other object whilst on a sledge—usually a tree. Falling off a sledge was far less harmful.

### DISCUSSION

Our observations clearly show that being hit by a sledge, or running into an inanimate object whilst on a sledge, is far more dangerous than an injury sustained falling off a sledge. What is not so clear is whether toboggans, trays or plastic sacks are the worst offenders. Also, is the slalem course at Brandon Hill, with its trees, benches and gaslamps, more lethal than the speedy downhill at Ashton Court?

Following this pilot study it is obvious that a comprehensive, double blind, randomised, crossover, prospective study is required to sort out these points. The public have the right to know.

### RECOMMENDATIONS

In the meantime we feel that all children under the age of 39 should be accompanied on the sledge by an adult. In addition at the next fall of snow a 'major accident' alert should be activated with a flying squad and mobile X-ray team dispatched to the black spots.

In summary, we conclude: "tobog or not to bog?", that is the question...

### ACKNOWLEDGEMENTS

Bill Shakespeare for the idea
Santa Claus for the toboggans
The Casualty Officers for requesting so many X-rays
Songs of Praise

